# Draft Genome Sequence of Oral Bacterium *Streptococcus mutans* JH1140

**DOI:** 10.1128/genomeA.00472-16

**Published:** 2016-06-02

**Authors:** Jerome Escano, Peng Deng, Shi-En Lu, Lief Smith

**Affiliations:** a Department of Biological Sciences, Texas A&M University, College Station, Texas, USA; b Department of Biochemistry, Molecular Biology, Entomology and Plant Pathology, Mississippi State University, Mississippi State, Mississippi, USA

## Abstract

*Streptococcus mutans* JH1140 is an oral bacterium known to produce the bacteriocin mutacin 1140, and the strain has been genetically engineered to combat dental caries. Here, we report the 2.0-Mb draft genome of *S. mutans* JH1140. This genome provides new insights into the strain’s superior colonization properties and its utility in replacement therapy.

## GENOME ANNOUNCEMENT

Dental caries is considered one of the most widespread chronic diseases afflicting the global population (1). Due to its production of lactic acid, the oral bacterium *Streptococcus mutans* is considered to be one of the major causes of dental caries (2). *S. mutans* JH1140 has been genetically modified to prevent dental caries (3). The technology is referred to as “replacement therapy” and stems from engineering the JH1140 strain to produce less acid. Gram-positive lactic acid bacteria, such as *S. mutans*, are known to produce bacteriocins (4). The ability of JH1140 to produce a potent bacteriocin enables the engineered replacement therapy strain BCS3-L1 to colonize the oral cavity of those inoculated. Mutacin 1140 is a bacteriocin produced by *S. mutans* JH1140. It belongs to a class of peptide antibiotics called lantibiotics (5). Mutacin 1140 has been shown to have a broad spectrum of activity against Gram-positive pathogens. Many bacteria are known to contain genes for more than one bacteriocin. Yet, many of these genes are expressed only under certain conditions, undermining efforts to isolate these bacteriocins. Assembly of the *S. mutans* JH1140 genome has helped identify novel bacteriocins and provided further understanding of the effector strain BCS3-L1’s superior colonization properties that facilitated its development as a replacement therapy strain.

*S. mutans* JH1140 was found to contain a single lactate dehydrogenase gene, which was predicted, given that this gene was mutated in the replacement therapy strain BCS3-L1. Aside from the mutacin 1140 operon, *S. mutans* JH1140 was found to contain two additional lantibiotic gene clusters. The first cluster contains similar genes for the two-component lantibiotic Smb (6). The other cluster contains three genes that encode a lantibiotic similar to nukacin (7). The nukacin-like gene cluster is the first instance of a lantibiotic gene cluster containing three repetitive genes that encode a similar lantibiotic. It is interesting to note that the Smb and nukacin-like lantibiotics have never been isolated from *S. mutans* JH1140.

Strain JH1140 was provided by the American Type Culture Collection (ATCC 55676). Genomic DNA from *S. mutans* JH1140 was isolated using an adapted protocol (8). Whole-genome sequencing was performed using the Illumina MiSeq 2 × 250-bp paired-end sequencer (Illumina, San Diego, CA) at the Genome Sequencing and Analysis Facility (University of Texas, Austin, TX). A total of 3,476,154 short reads (1 Gb) were *de novo* assembled using the ABySS version 1.9.0 and DNAstar SeqMan NGen version 12 softwares. The assembled genome had 525× genome coverage. The draft *S. mutans* JH1140 genome contains 24 contigs and has an approximate size of 2 Mb, with a G+C content of 36.6%. Gene annotation was performed by the NCBI Prokaryotic Genome Annotation Pipeline using the GeneMarkS+ version 3.1 software. The genome annotation consisted of 1,901 protein-coding genes, with 379 of them being hypothetical protein-coding genes. There were 61 RNA genes predicted in the genome annotation, consisting of genes encoding 50 tRNAs and 7 rRNAs.

### Nucleotide sequence accession number.

This draft genome sequence has been deposited at GenBank under the accession no. LTAK00000000.
